# COVID-19 vaccination in the mass vaccination center: clinical practice and effectiveness analysis

**DOI:** 10.3389/fpubh.2023.1072883

**Published:** 2023-06-15

**Authors:** Jie Fan, Ling Zhu, Xiaohua Wu, Chunyu Luo, Ailong Huang, Wei Wang

**Affiliations:** ^1^School of Public Health, Chongqing Medical University, Chongqing, China; ^2^Nanan District Center for Disease Control and Prevention, Chongqing, China; ^3^Key Laboratory of Molecular Biology on Infectious Diseases, Ministry of Education, Chongqing Medical University, Chongqing, China

**Keywords:** COVID-19, vaccine, vaccination experience, adverse events following immunization, mass vaccination center

## Abstract

**Objectives:**

Mass vaccination campaigns can rapidly increase the vaccination rate for the COVID-19 vaccine, the establishment of mass vaccination centers is indispensable. At the beginning of March 2021, China began to carry out COVID-19 vaccination activities nationwide. Here, we aimed to evaluate the criteria established by mass vaccination centers, COVID-19 vaccination experience, the incidence of adverse events following immunization and opinions.

**Methods:**

We describe the layout and functioning of Nan’an District mass vaccination center, the working mechanism, experience and effectiveness. Distribution of COVID-19 vaccine vaccination and adverse events following immunization reported in the mass vaccination center of Nan’an District were evaluated.

**Results:**

From March 26, 2021 to April 28, 2022, the mass vaccination center has inoculated about 381,364 doses of COVID-19 vaccine to the population. The study found that the incidence of adverse events following immunization (AEFI) was very low (1.04/100000). The chances of having AEFI were significantly higher in COVID-19 vaccine (CHO cell) than COVID-19 vaccine (Vero cell).

**Conclusion:**

The mass vaccination center was running successfully. It was effective and safe, providing vaccination services and increasing COVID-19 vaccination rates among the population. The experience of the mass vaccination center for COVID-19 in China can provide a reference for other countries and regions to carry out COVID-19 vaccination.

## Introduction

Coronavirus disease (COVID-19) has spread in almost all countries, and the pandemic remains aggressively spreading (1). According to the World Health Organization (WHO), as of September 7, 2022, more than 600 million confirmed cases of COVID-19 worldwide, including more than 6.4 million deaths. COVID-19 is a public health problem of global concern, posing a severe threat to, the environment, health and economic recovery (2, 3). During the pandemic, all countries have adopted prevention strategies to curb the spread of COVID-19, including tracking contacts, lockdown measures, keeping social distance, proper sanitation and wearing masks (4, 5). Many countries have strengthened border control and actively monitored to detect and isolate imported cases quickly (6).

Recently, many countries have canceled prevention and control measures, relaxed entry prevention policies, and no longer implemented mandatory wearing of masks. It is worth mentioning that vaccination is still advocated (7). Since the COVID-19 epidemic, China has always adhered to the “people-oriented and life oriented,” and promoted epidemic prevention and control with high-quality strength of the whole country. It has also achieved the effect of rapid control of the epidemic and rapid economic recovery and development. Since August 2021, China has always adopted a dynamic zero-COVID-19 strategy in response to the COVID-19 epidemic (8). The “dynamic zero-COVID-19” strategy follows the prevention and control strategy of “external prevention input, internal prevention rebound.” When COVID-19 cases occur locally, precise prevention and control measures should be taken to quickly cut off the epidemic transmission chain so that each epidemic situation can be terminated (9, 10). Since January 8, 2023, the management of COVID-19 in China has been adjusted to the management of category B infectious diseases, and vaccination and personal protection are still advocated.

Currently, there is no effective antiviral drug to treat COVID-19, but the existing medications for treating other viral diseases have improved the rehabilitation of severe patients (11–13). During the COVID-19 pandemic, an effective mass vaccination campaign was essential to control the virus. Vaccination is a central strategy for mitigating and controlling the COVID-19 pandemic (14). Up to September 2022, more than 12 billion doses of vaccine have been inoculated (data from WHO). Mass vaccination creates an important prerequisite for the adjustment of COVID-19 prevention and control strategies in the future. According to WHO, the first mass vaccination plan will be launched in early December 2020 (15). At the beginning of March 2021, China began to carry out COVID-19 vaccination activities nationwide. Mass vaccination centers for COVID-19 vaccines have been established all over the country to gradually increase residents’ vaccination rates. As of September 7, 2022, a total of 34,3,396 million doses of the COVID-19 vaccine had been reported in China (16). Different countries may have different patterns of large-scale COVID-19 vaccination. In Italy, local and state health authorities cooperate with private units to establish temporary vaccination sites by changing the use of buildings (14). Maritza Suarez et al. reported how to carry out mass vaccination of COVID-19 in an academic health center at the University of Miami (17).

Vaccination is an essential means to maintain public safety and interrupt the spread of COVID-19, as well as an essential way to protect people’s life and health. “large-scale vaccination” is a valuable experience summed up in epidemic prevention and control. Large-scale vaccination can rapidly increase the coverage of the population and form a community immunity barrier. Clinical practice and effectiveness analysis of the COVID-19 vaccination the mass vaccination center can provide a reference for emergency vaccination of emerging infectious diseases. However, countries around the world face problems and challenges in planning and implementing vaccination strategies, and the operation of mass vaccination centers needs further study (18). In this study, we aimed to evaluate the criteria established by mass vaccination centers in Chongqing China, COVID-19 vaccination experience, and post-vaccination adverse events and opinions, to provide a reference for other countries to establish mass vaccination centers.

## Materials and methods

### The structure of mass vaccination center

**Figure 1:**
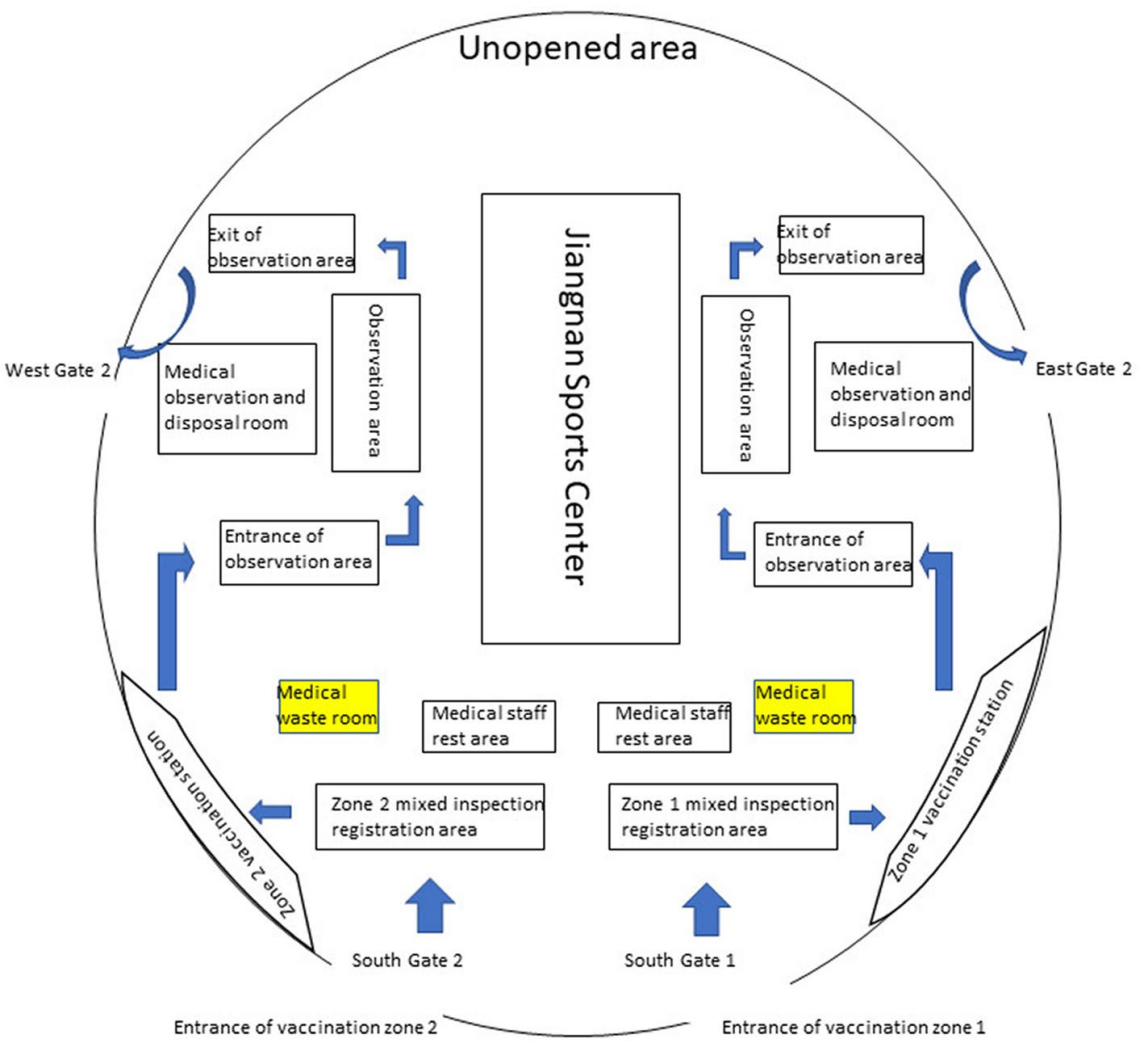
The floor plan of Nan’an District mass vaccination center.

Nan’an District of Chongqing has an area of 262.43 square kilometers, a permanent population of 1.2 million, and an urbanization rate of 97.8%. Nan’an District mass vaccination center is located in the Nan’an District Jiangnan Sports Center, which can hold more than 3,500 people. As shown in Figure 1, the mass vaccination center is divided into vaccination sites A and B; vaccination sites are divided into waiting areas, Pre-inspection areas, registration areas, inoculation areas, cold chain areas, observation areas and rescue areas. Each functional area has apparent signs. The workflow of vaccination, vaccination contraindications, immunization procedures, and possible adverse reactions was pasted in visible places. There is only one road, one-way, from entering the vaccination sites to leaving after the vaccination is completed. The purpose of this is to avoid cross-infection.

### Equipment and facilities

As shown in Table 1 and Figure 1, The Jiangnan Sports Center in Nan’an District was transformed, and the critical functional area for COVID-19 vaccination was established. Vaccination sites are equipped for epidemic prevention and control, vaccine storage Cold-chain equipment and facilities, injection equipment, and vaccines commensurate with the number of vaccinators. The specific quantity of medicines, instruments and first-aid equipment and facilities should be estimated according to the average daily Determination of inoculation quantity.

**Table 1:** Equipment and facilities configuration of the mass vaccination center.

Area	Equipment and facilitie
Entrance	Forehead thermometer, hands-free disinfectant, queuing machine
Waiting areas	COVID-19 vaccination campaign materials, provide a sufficient number of seats for the recipients, equipped with heating and cooling equipment
Registration area	Computer, informed verification device, code scanning gun (can scan QR code), hand sanitizer, body temperature gun, Precautions for COVID-19 vaccination, ID card reader (optional)
Inoculation area	Computer, one verification device (optional), code scanning gun, printer, hand sanitizer, freezer or freezer package (ice sheet, thermometer, vaccine shall not be in direct contact with ice sheet), temperature record sheet, disinfection cotton swabs, 75% alcohol, syringes, treatment tray, medical waste bucket, sharp instrument box, household trash can, monitoring equipment
Cold chain area	Medical refrigerator, monitoring equipment, thermometer, temperature record book
Observation and rescue area	Warm water, clock, monitoring equipment, precautions after COVID-19 vaccination
Disposal area of suspected abnormal vaccination reactions	All kinds of emergency medicine:observation bed, epinephrine hydrochloride (1 mg/each), norepinephrine bistartrate injection (1 mg/each) (2 mg/branch), Dixixi injection (10 mg/branch), amiodarone hydrochloride injection (amiodarone) (150 mg/branch), 50% glucose injection (20 mL/branch), Diphenhydramine hydrochloride injection (20 mg/branch), dexamethasone sodium phosphate injection (5 mg/branch), 0. 9% sodium chloride injection (500 mL/bag), 5% glucose injection (100 mL/bag); Simple respirator (balloon mask), defibrillation apparatus or AED1 mind, multifunctional monitor, the electrocardiogram machine, a portable oxygen tank, nasal catheter oxygen mask, stethoscope, and several spatula, pupil pen mind, oropharynx ventilation tube, fast blood glucose meter, endotracheal intubation, blood sugar test paper 1 set of laryngoscopes, laryngoscope blades (large, medium, and small), oropharyngeal airway, 1 set of negative pressure suction device, sputum suction tubes, endotracheal intubation catheters, endotracheal intubation guide wire, vomiting kits etc.
Others	Disposable surgical mask, protective suit, quick hand disinfectant, disposable medical hat, gloves, disposable face screen, etc.

### Personnel arrangements and training

The staffing profile is shown in Table 2. Based on the adequate assessment of existing vaccination capacity, based on the number of appointments on the day of the reasonable allocation of staff. The Nan’an District Center for Disease Control and Prevention provided special training on the Emergency Use of the COVID-19 Vaccine to the staff of the mass vaccination center,training on the Novel Coronavirus Vaccine Treatment Protocol for Common Suspected Coronavirus Vaccination Reactions.

**Table 2:** Description of employee responsibilities in mass vaccination center.

post	number	duty
General leader	1	Coordinate the work of the mass vaccination center
Pre-check registration personnel	12	Vaccination registration, pre-screening, etc
Vaccination personnel	10	Standardized implementation of vaccination
Medical rescuing	2	During the period of vaccination, they were kept in the observation area for touring observation. During the period of vaccination, they were kept in the observation area for touring observation. If discomfort was found, The patients were guided to the observation room in time and treated in a standardized manner
Network maintenance	1	System maintenance and network maintenance
Quality control officer	2	Quality control of inoculation process
Volunteer	Several	Maintain order

### Source and use of COVID-19 vaccines

Types of COVID-19 vaccines: (a)The inactivated COVID-19 vaccine (Vero cells) was produced by Sinopharm China Biologic Beijing Institute of Biological Products Co., Ltd. and Beijing Kexing Zhongwei Biotechnology Co., Ltd. (b)The recombinant subunit Novel COVID-19 vaccine approved for emergency use is the Recombinant Novel coronavirus vaccine (CHO cell) produced by Anhui Zhifei Longkema Biopharmaceutical Co., Ltd. (Zhifei Longkema).

The recommended dosage: (a) Sinovac-CoronaVac/Sinopharm COVID-19 vaccine: 2 doses (0.5 mL) given intramuscularly. The second dose shall be administered 21 days after the first dose of vaccine. (b) recombinant subunit Novel COVID-19 vaccine (ZhifeiLongcom, China): 3 doses (0.5 mL) given intramuscularly, each dose should be given for a month.

Applicable object: (a) Sinovac-CoronaVac/Sinopharm COVID-19 vaccine: people over 3 years old. (b) recombinant subunit Novel COVID-19 vaccine (ZhifeiLongcom, China): people over 18 years old.

### The implementation phase of COVID-19 vaccination

The principle of vaccination is as follows: according to the prevention and control strategies of external prevention of import and internal prevention of rebound and the requirements of the epidemic prevention and control situation, prioritize and steadily promote vaccination.

**Phase 1:** The highest-risk populations, including such as those engaged in import cold chain, hip pilotage, port quarantine, aviation and air service, public transport, medical disease control, fresh markets, and those who go to work or study in medium and high-risk countries or regions.

**Phase 2:** People aged 18–59 years without vaccination contraindications.

**Phase 3:** People aged >60 years and 12–17 years without vaccination contraindications.

**Phase 4:** People aged 3–11 years without vaccination contraindications.

### Surveillance and reporting of adverse events following immunization

#### Range of AEFI report

Any reaction or event suspected to be related to COVID-19 vaccination should be reported, especially severe or mass AEFI. No online reporting was required for expected common, minor reactions, such as mild fever, local pain, or redness. The network report should be conducted in the case of fever ≥38.5°C, redness and swelling, or induration diameter > 2.5 cm.

#### Reporting procedure

Healthcare workers may report the occurrence of AEFI within 30 min after vaccination. Subsequently, recipients or their guardians should actively report AEFI to the vaccination unit. For reportable AEFI or any other AEFI deemed to be reportable, the mass vaccination centers should report it to the China’s disease prevention and control information system within 48 h after detection. In addition, when an AEFI is suspected to be related to COVID-19 vaccination, such as death, severe disability, or mass adverse events following immunization, it should be reported to the national system within 2 h.

#### Investigation and diagnosis

Nan’an District Center for Disease Control and Prevention verified and evaluated the suspected abnormal vaccination reaction of the novel coronavirus vaccine. Case investigation is required if it is classified as a severe AEFI, and for the non-seriousAEFI, it is generally unnecessary to carry out a case investigation. The CDC will review and classify it according to the assessment.

### Prevention and control measures

To prevent nucleic acid contamination caused by COVID-19 vaccine “False positive” event, specific measures: (a) Personnel should operate in a standardized manner. Inoculation nurses must to wear protective clothing, caps, medical protective masks, face screens, gloves and boot covers. (b) Enhance environmental management. The mass vaccination center Often disinfect and wipe, and make corresponding records. Disinfection before daily vaccination and disinfection of the environment after vaccination. Chlorine-containing disinfectant containing 250 ~ 500 mg/L effective chlorine or 1% hydrogen peroxide can be used for spraying, wiping or mopping disinfection for 20 ~ 30 min. (c) Enhance medical waste management. Dispose of disposable syringes, self-destructing syringes and other medical wastes after use in strict accordance with the Regulations on the Management of Medical Wastes. After mass vaccination, the mass vaccination center collects all medical wastes and uniformly collects them for disposal. The cotton swabs/cotton balls pressed on the vaccination site shall not be discarded or taken away from the site at will.

### Statistical analysis

We used Microsoft Excel 2019 and statistical analysis software (SPSS 26.0 version) for statistical analysis, and used online websites^1^ to draw pictures. AEFI cases are derived from China’s disease prevention and control information system. Descriptive statistics method is adopted for the basic situation of mass vaccination centers. The categorical variable analysis adopts the *χ*^2^ test or Fisher exact test. *p*-values less than 0.05 are considered statistically significant.

## Results

### Standardize the vaccination process

**Figure 2:**
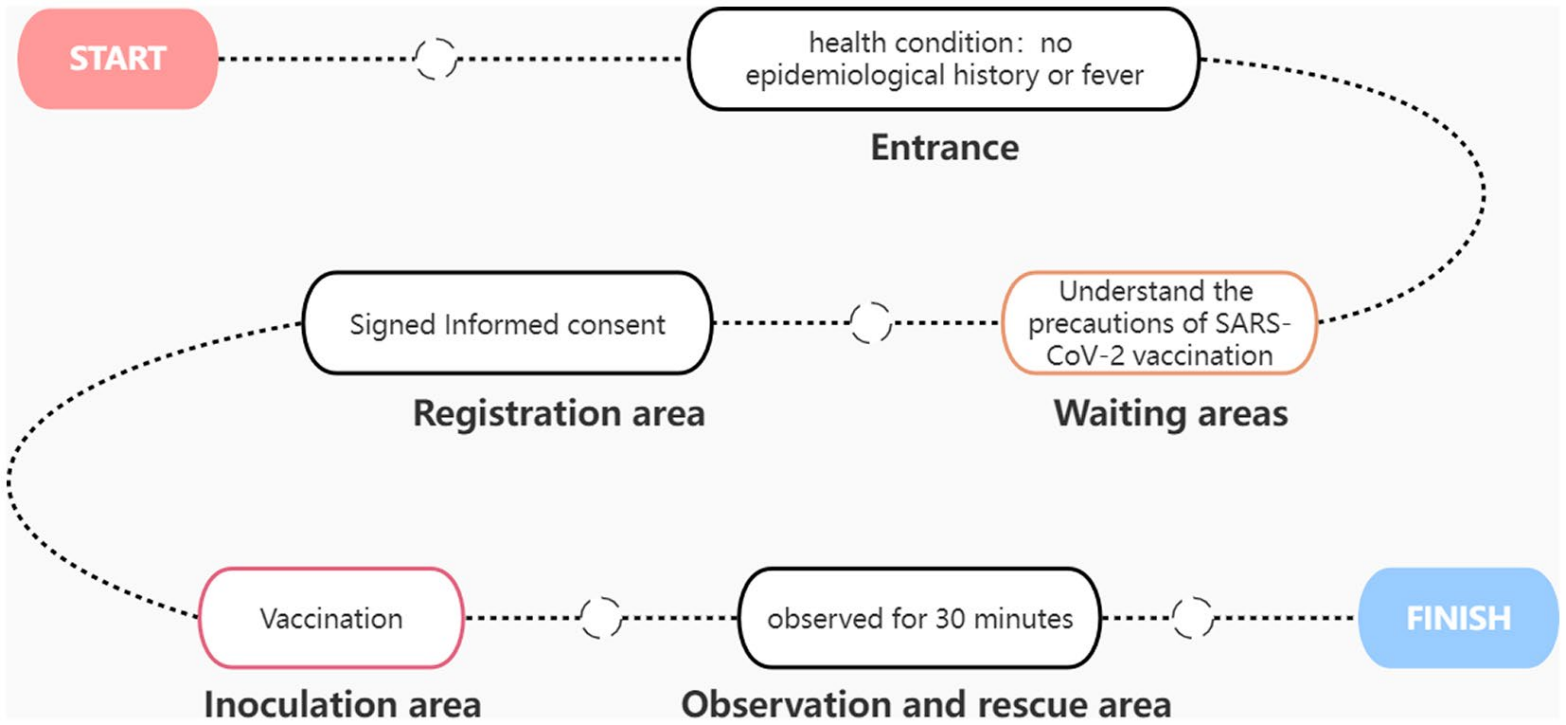
Process of SARS CoV-2 vaccination.

A simple SARS-CoV-2 vaccination process is shown in Figure 2. At the entrance, the staff inquired about the recipient’s epidemiological history and fever history. Only residents without epidemiological history and fever history can enter the waiting area. Each waiting area is equipped with medical staff to provide services and receive consultations at any time. After waiting for a few minutes, the vaccinators enter the pre-examination registration area, where they must sign an informed consent form stating the occasional discomfort that may occur. After registration, the vaccine recipients arrive at the vaccination area. Each injection table is equipped with a desktop refrigerator for storing vaccines. After vaccination, the vaccinated person shall be transferred to the observation area. The observation area shall be equipped with at least two medical personnel to observe the health status of the recipients at any time. After 30 min of observation, no ill vaccinators can leave. The first aid room and ambulance are equipped with first-aid medicine, equipment and medical personnel. The ambulance is parked at the exit of the room. If any allergic reaction is observed, the recipient will receive treatment first and be transferred to the general hospital in time.

### Distribution of COVID-19 vaccine vaccination

**Figure 3:**
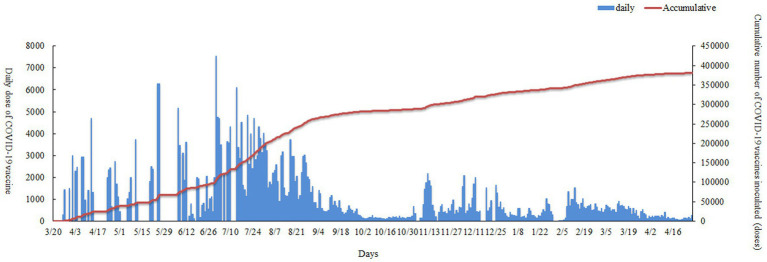
Nan’an District mass vaccination center administration vaccine trend.

From March 26 2021 to April 28 2022, 381,364 vaccine doses were administered by Nan’an District mass vaccination center to the population: CoranaVac (vero cell) 159,047 doses, Sinopharm (Vero cell) 130,118 doses and Recombinant novel coronacirus (CHO cell) 92,199 doses. As shown in Table 3, Vaccination sites A (Nanan District Maternal and Child Health Hospital) and vaccination sites B (Longmen Hao Street Community Hospital) had similar numbers of COVID-19 vaccinations. Nan’an District mass vaccination center administration vaccine trend as shown in Figure 3. The red line shows the cumulative inoculum doses. The blue bar chart shows the number of COVID-19 vaccine vaccinations per day. A total of 7,550 doses were administered at the mass vaccination center on July 1, 2021, which was 5.46 times the average dose administered at other vaccination sites. There was a wide variation in daily COVID-19 vaccine doses. The maximum amount in a single day was over 7,500, and the minimum dose was 0. The daily dose administered is mainly influenced by local vaccination policies and the timely availability of vaccines. To rationalize vaccination resources, the mass vaccination center will adjust the number of staff and increase or decrease the number of vaccination stations daily.

**Table 3:** Distribution of COVID-19 vaccine vaccination.

Variables	Vaccine type			Total
	CoranaVac (vero cell)	Sinopharm (vero cell)	Recombinant novel coronacirus (CHO cell)	
Vaccination sites A	78,551	64,190	46,042	188,783
Vaccination sites B	80,496	65,928	46,157	192,581
Total	159,047	130,118	92,199	381,364

### Description of people with adverse reactions to COVID-19 vaccination

In the case of AEFI, the staff shall enter the relevant information into the national system following the National Work Plan for Monitoring Suspected Abnormal Vaccination Reactions within 48 h. If serious AEFI occurs, it must be reported within 2 h. In this study, only four people reported common AEFI after receiving the COVID-19 vaccine. The incidence of adverse events following immunization (AEFI) was very low (1.04/100000). Specific information, as shown in Table 4, the most common local AEFI is injection site pain. This study estimated the risk of AEFI according to the recipient’s age, sex and type of COVID-19 vaccine who received the COVID-19 vaccine. We observed no statistical difference in the occurrence of AEFI in terms of age, sex, etc., except for the type of vaccine. Compared with the Vero cell vaccine, the CHO cell vaccine has a higher tendency to produce AEFI (Table 5).

**Table 4:** Description of people with adverse reactions to COVID-19 vaccination.

Number	Sex	Age	Vaccine type	Symptom	Definitive diagnosis
1	Female	70	CHO	Abnormal redness at the injection site	Common vaccination reactions
2	Male	23	VERO	Fever	Common vaccination reactions
3	Female	37	CHO	Abnormal redness at the injection site	Common vaccination reactions
4	Male	29	CHO	Skin eruption	Anaphylactic rash

**Table 5:** Risk estimations of adverse events after the dose of the COVID-19 vaccine among the respondents.

Varibales	Adverse events after the dose of COVID-19 vaccine		*P-value*
	Yes (*N* = 4)	No (*N* = 381,360)	
Sex			
Male	1	121,932	0.617
Female	3	259,428	
Age			
Below 59	3	347,819	0.308
60 or above	1	33,541	
Vaccine type			
Vero cell	1	289,165	**0.046** ^*^
CHO cell	3	92,196	
Vaccine type^(a)^			
Vero cell	1	243,678	0.066
CHO cell	3	92,196	

Vaccine type^(a)^: we excluded recipients under 18 years old. *P < 0.05 was considered significant.

## Discussion

Vaccination is one of the most effective, safe and cost-effective public health interventions widely used (19). The objective of COVID-19 vaccination is to protect susceptible persons, increase coverage rate of population vaccination, establish a community immunity barrier, and eventually eliminate the virus (20). The risk of serious diseases caused by COVID-19 increases with age, and AEFI causing serious death is very rare. Therefore, we should encourage COVID-19 vaccination, especially for the older adult, to prevent virus infection. Implementing mass vaccination campaigns is a complex decision-making process, depending on the country’s specific situation. In this study, the mass vaccination centre has been very successful. The success of these efforts depends mainly on the collaboration and coordination between the local government departments, the Health Commission, the sub-district offices, and community workers and volunteers. Nan’an District CDC has provided professional technical guidance for implementing mass vaccination, including vaccination site construction standards, vaccination specifications, personal protection and strengthening health work.

Generally, mass vaccination centers are established in places with convenient transportation, a concentrated population and good ventilation. Nan’an District mass vaccination center is located in the Nan’an District Jiangnan Sports Center, an area with convenient traffic and concentrated residents. Residents can get here quickly by public transportation, such as buses and subways. While we are opening mass vaccination centers, routine vaccination clinics still carry out vaccination services, and the older adults and children can choose other places nearby according to their specific circumstances. In actual work, if we meet older adults with mobility problems, we will provide on-site vaccination services. The public is worried that the mass vaccination centers can potentially increase the risk of cross-infection among people. We have taken a series of measures to avoid cross-infection. Firstly, masks are mandatory for all, the staff inquired about the recipient’s epidemiological history and fever history at the entrance. Only residents without epidemiological history and fever history can enter the waiting area. Secondly, Disinfection before daily vaccination and Disinfection of the environment after immunization. Thirdly, if there is a COVID-19 outbreak in the local area, we will temporarily close the mass vaccination centers. So far, there has been no cross-infection in the mass vaccination centers.

The public is also worried about the safety and effectiveness of COVID-19 vaccine (21). Therefore, it is essential to evaluate and monitor the adverse reactions after COVID-19 vaccination (22, 23). As of May 30, 2022, in China, 238,215 adverse events after vaccination had been reported, with an overall reported incidence of 7.45/100000 (24). In the mass vaccination center, we observed that the incidence of adverse events following immunization was very low, and no severe AEFI occurred. Only four respondents reported common AEFI after the doses, with an overall reported incidence of 1.04/100000. Recipients should be encouraged to actively report adverse events to obtain as much information as possible to analyze and improve activities continuously. This study analyzed the impact of age, sex and type of vaccination on the incidence of adverse events. Dipu T. S. et al. reported the incidence of AEFI was observed significantly high among females, our study did not observe significant association between the incidence of AEFI and gender (25). Researchers were more concerned about the incidence of adverse events among the older adults, so we divided the population into people over 60 years old and under 60 years old when analyzing age factors. The statistical results showed no statistically significant difference in the incidence of adverse events among different ages after COVID-19 vaccination. The American the Centers for Disease Control and Prevention (CDC) reported the proportion of minor side effects in the older adults were similar to those in other adult populations, this was consistent with the results of our study. From the data point of view, the number of inactivated vaccines inoculated is far greater than that of CHO vaccines. Our study demonstrated lower AEFI among the Vero cell vaccine and CHO cell vaccine (*p* = 0.046). Considering that two vaccines were used for vaccination, the age of the people vaccinated with these two vaccines was inconsistent. Vero cell vaccine was over 3 years old, and the other was over 18. There were no adverse reactions among people under 18 years old. So we excluded recipients under 18 years old, and the new statistical results showed no statistically significant difference in the incidence of adverse events among different COVID-19 Vaccine type vaccination (Table 5). The number of AEFI is too small, and we need more samples to analyze whether the type of vaccine affects the incidence of AEFI.

## Limitations

This study has several limitations. Firstly, there is a significant staff turnover in mass vaccination center, and it takes time for new teams to become familiar with their job responsibilities and content, which may inevitably lead to omissions. Secondly, in the actual vaccination process, sometimes the prediction of the number of recipients on the same day was inaccurate, which led to reduced vaccination efficiency and other problems. Thirdly, the reported number of adverse events after immunization may be less than the actual number of events that occurred in mass vaccination centers, which may be due to insufficient voluntary reporting. The recipients feel that injection pain or fever are common vaccination reactions and have not reported them in time. The above problems need to be gradually improved on the future practice process.

## Conclusion

In summary, the mass vaccination center was run successfully. It was effective and safe, providing vaccination services and increased COVID-19 vaccination rates among the population. The experience of the Mass Vaccination Center has provided a successful working mechanism example for the global prevention and control of COVID-19, and other countries and regions can learn from it when carrying out mass vaccination.

## Data availability statement

The raw data supporting the conclusions of this article will be made available by the authors, without undue reservation.

## Funding

This research was supported by the Science and Technology Commission joint medical research Project Foundation of Nan’an district (No. 2021–17).

## Author contributions

JF, AH and WW completed the research design. JF analyzed the data and wrote the first draft. JF, LZ, XW, and CL collected data. AH and WW commented on all sections of the paper and edited the final manuscript. All authors read and approved the final manuscript, contributed to this study, and commented on the previous version.

## Conflict of interest

The authors declare that the research was conducted in the absence of any commercial or financial relationships that could be construed as a potential conflict of interest.

## Publisher’s note

All claims expressed in this article are solely those of the authors and do not necessarily represent those of their affiliated organizations, or those of the publisher, the editors and the reviewers. Any product that may be evaluated in this article, or claim that may be made by its manufacturer, is not guaranteed or endorsed by the publisher.

## Footnotes

^1^ www.processon.com
